# SiOC Screens with Aligned and Adjustable Pore Structure for Screen Channel Liquid Acquisition Device

**DOI:** 10.3390/ma16031063

**Published:** 2023-01-25

**Authors:** Pedro Henrique da Rosa Braun, Prithvi Shukla, Kurosch Rezwan, Michael Dreyer, Michaela Wilhelm

**Affiliations:** 1 Advanced Ceramics, University of Bremen, Am Biologischen Garten 2, IW3, 28359 Bremen, Germany; 2 Center of Applied Space Technology and Microgravity (ZARM), Department of Fluid Mechanics, Faculty of Production Engineering—Mechanical Engineering and Process Engineering, University of Bremen, Am Fallturm 2, 28359 Bremen, Germany; 3 MAPEX—Center for Materials and Processes, University of Bremen, Am Fallturm 1, 28359 Bremen, Germany

**Keywords:** SiOC screens, unidirectional freeze-casting, bubble point, polymer-derived ceramic, gas–liquid phase separation

## Abstract

The development of porous ceramic screens with high chemical stability, low density, and thermal conductivity can lead to promising screen channel liquid acquisition devices (SC-LADs) for propellant management under microgravity conditions in the future. Therefore, SiOC screens with aligned pores were fabricated via freeze-casting and applied as a SC-LAD. The pore window sizes and open porosity varied from 6 µm to 43 µm and 65% or 79%, depending on the freezing temperature or the solid loading, respectively. The pore window size distributions and bubble point tests indicate crack-free screens. On the one hand, SC-LADs with an open porosity of 79% removed gas-free liquid up to a volumetric flow rate of 4 mL s^−1^. On the other hand, SC-LADs with an open porosity of 65% were limited to 2 mL s^−1^ as the pressure drop across these screens was relatively higher. SC-LADs with the same open porosity but smaller pore window sizes showed a higher pressure drop across the screen and bubble ingestion at higher values of effective screen area when increasing the applied removal volumetric flow rate. The removed liquid from the SC-LADs was particle-free, thus representing a potential for applications in a harsh chemical environment or broad-range temperatures.

## 1. Introduction

Phase separation is a critical task for fluid management in many space applications. One example is a gas-free liquid propellant supply from the propellant tank to the engine or the refueling of spacecraft from a supply tank in microgravity [1]. In the absence of gravity, capillary forces become the dominant forces controlling the liquid–gas interface inside the tank. Porous media play an important role in phase-separation applications. Based on capillary action, a saturated porous medium can act as a barrier for gas ingestion under a specific set of conditions, and ensure a gas-free supply of propellant. A screen channel liquid acquisition device (SC-LAD) is a type of liquid acquisition device that works according to the principles mentioned above [2]. A typical SC-LAD is defined as a closed channel with three solid walls and one porous wall. Liquid can enter into the channel through the porous screen but the entry of the gaseous phase will be blocked as long as the total pressure drop across the porous screen is less than the bubble point pressure. The bubble point of the porous screen is the most important performance evaluator for a SC-LAD. It is the maximum pressure difference between the liquid and gas phases that the porous screen can withstand. It depends on parameters such as the surface tension of the liquid, the contact angle between the liquid and the solid, and the biggest pore window size, known as the bubble point diameter of the porous screen [3,4,5]. Besides the bubble point pressure, another important parameter affecting SC-LAD performance is the flow-through-screen pressure drop. It takes into account the pressure loss that occurs as the liquid moves across the wetted area of the porous screen [6]. It depends on the properties of the liquid and the screen, as well as on the superficial velocity in the same direction of the liquid flow. The selection of optimal porous screens for a particular application/mission depends on the environmental conditions along with the required outflow [1,7]. In general, a porous screen with a high bubble point is desirable which means that a smaller bubble point diameter is required. On the other hand, small pores lead to an increase in the flow-through-screen pressure drop which is not desirable and limits the permissible outflow rate of the liquid. Thus, an optimum balance of properties of porous screens is required to ensure a satisfactory performance for the desired application. A SC-LAD is generally designed using metallic screens as porous material. Metallic screen-integrated SC-LADs have proven flight heritage over the last five decades in the space industry. The metallic screens are woven screens with wires usually made of aluminum, titanium, or a stainless-steel alloy. The geometric properties of the screen depend on the weave pattern and the material of the wire [8,9,10]. The knowledge of the geometry of the metallic screen is sufficient to calculate geometric properties such as the pore diameter, porosity, thickness of the screen, and surface-to-volume ratio using the proper equations [11,12]. Numerous experimental and simulated data can be found in the literature where metallic screens have been tested under Earth gravity conditions and in microgravity environments [13,14,15,16,17]. After two initial papers dealing with the applicability of porous SiOC monoliths for isothermal/cryogenic wicking [18,19], there are, to the authors’ knowledge, no data reported on the further development of these materials into crack-free SiOC as SC-LADs, the correlation of experimental results with the pore structure, or an analytical solution. The absence of the application of SiOC screens in relevant engineering fields of application can be attributed to the natural brittleness of porous ceramics and the challenge of producing crackless porous ceramics that allow particle-free phase separation. Porous ceramics can be applied in applications such as catalyst support, energy harvesting, or filtration [20,21,22] and may offer some advantages compared to metallic screens such as chemical and thermal stability, relatively lower density, and thermal conductivity [23,24]. Relatively low values of thermal conductivity between 0.2 W m^−1^ K^−1^ and 2.0 W m^−1^ K^−1^ were reported for porous and non-porous SiOC, respectively, in a temperature range from 77 K to 1400 K [23,25]. These advantages allow the creation of corrosion-resistant screens, a system with reduced mass, and components that are thermally stable enough to avoid the boil-off of propellants under cryogenic conditions. Polymer-derived ceramics are an alternative class of ceramic materials that possess some material and process-related advantages compared to oxidic ceramics such as Al_2_O_3_ or TiO_2_. By shaping, cross-linking, and pyrolysis under an inert gas atmosphere, preceramic polymers can be converted into ceramics at relatively lower temperatures (1000–1200 °C) [26,27,28]. Surface characteristics such as hydrophilicity and specific surface area (i.e., microporosity) can be widely adjusted based on the starting composition and pyrolysis temperature [29]. Additionally, the pore structure of the ceramic screen can be tailored when using shaping methods such as replica or direct foaming [30]. However, these conventional processing techniques offer a limited range of achievable pore morphologies, pore directionality, and pore window size distribution. Solution-based freeze-casting arises as a flexible sacrificial templating method that allows the creation of a wider range of pore structures [15,31]. This process starts with dissolving a preceramic polymer in an organic solvent followed by freezing this polymer solution. With the onset of freezing, solvent crystals form and are separated from the preceramic polymer (i.e., solid loading) by thermal-induced phase separation. Later, the solidified crystals are sublimated from the porous network and form the open porosity of the sample. Properties related to the pressure drop across the SC-LAD such as the pore window size distribution and open porosity can be tuned from 1 µm to 100 µm and from 20% to 90%, depending on the freezing temperature of the polymer solution and its polymer concentration (i.e., solid loading), respectively [15,32]. Furthermore, by creating a temperature gradient during the freezing of the polymer solution, an aligned pore network of screens can be developed for a lower pressure drop during mass transport. By choosing different solvents, one can develop dendritic, lamellar, prismatic, or honeycomb-like pore morphologies [33,34]. Previous studies have depicted the influence of different pore morphologies of SiOC ceramics on wicking transport [19]. In addition to the ability to transport liquids, the dendritic pore structure exhibits increased mechanical stability [35]. In this study, we apply a ceramic screen as a SC-LAD. Screens with an aligned and dendritic pore structure were created from solution-based freeze-casting of preceramic polymers. Pore window size distribution and open porosity were tailored using different freezing temperatures and solid loading. The combined characterization of the pore structure with SEM images, the mercury (Hg) intrusion method, and bubble point tests simultaneously allowed us to check whether the screens produced were crack-free and homogenous. Correlating data from gas–liquid phase separation with the pore structure allows us to understand if the relationship between the pressure drop, pore window size, and open porosity hold for thicker screens with a broader pore window size distribution (i.e., porous SiOC screens). In summary, this study provides novel information on the first-time fabrication of a crackless SiOC screen with special pore geometry, its use in a SC-LAD, and a more comprehensive study on the correlation between the pore structure of a ceramic screen and its phase-separation capability as a SC-LAD.

## 2. Experimental Section

### 2.1. Materials

The SiOC screens were prepared by unidirectional solution-based freeze-casting using a solid loading composed of 99 mol% of polymethyl siloxane (Silres^®^ MK, Wacker Chemie AG, Munich, Germany, molar mass *M* = 70.29 g mol^−1^) as preceramic polymer and 1 mol% (3-aminopropyl) triethoxysilane (APTES, ABCR GmbH, Karlsruhe, Germany, *M* = 221.37 g mol^−1^) as cross-linking agent. These components formed the solid loading, accounting for 20 wt% or 30 wt% of the total mass of the polymer solution. The remaining mass fraction of the polymeric solution (80 wt% or 70 wt%) consisted of the template media (liquid solvent) which was cyclohexane (CH, >99%, *ρ* = 778 kg/m^−3^, Sigma-Aldrich Chemie GmbH, Hamburg, Germany). All materials were used without further treatment.

### 2.2. Manufacture of SiOC Screens

**Figure 1:**
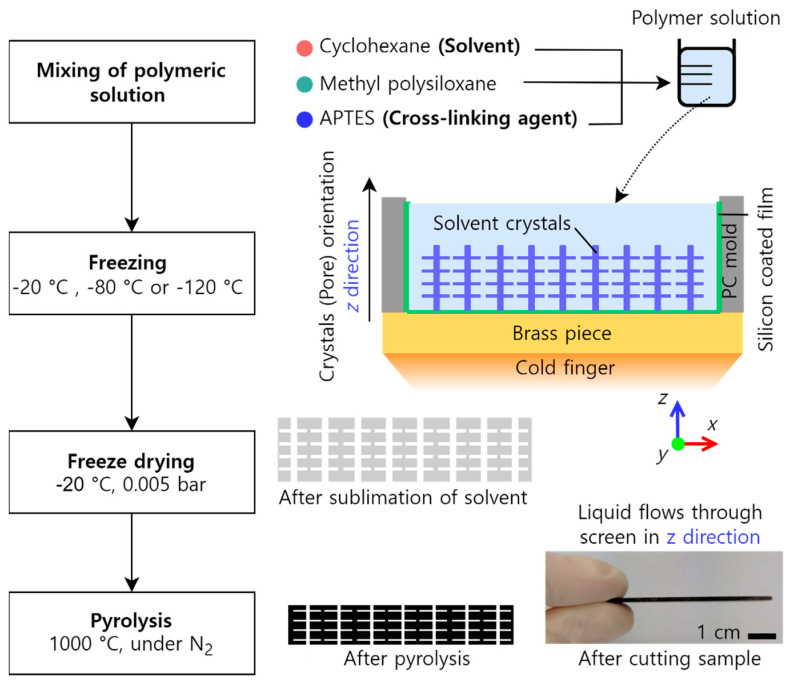
Flowchart of the manufacturing route via freeze−casting used in this study.

The complete manufacturing route of the screens via the freeze-casting process is shown in Figure 1. The preparation of the polymer solution started with dissolving the preceramic polymer MK in the solvent CH under vigorous stirring at room temperature. Subsequently, the cross-linking agent APTES was added to the mixture forming the polymeric solution. The solution was then poured into the sampling mold and degassed at 0.3 bar for 15 s to remove any gas bubbles created during the stirring or casting procedure. The freezing of the solution started in the sampling mold after the mold with the cast solution was exchanged for a control mold, which was used on the cold finger to set the temperature. In this way, ice formation on the surface of the brass piece was avoided during the stabilization of the freezing temperature. The molds consisted of an upper part made of polycarbonate (height of 20 mm) and a lower part made of solid brass, which was in contact with the top of the cold finger of copper (Figure 1). A silicone-coated polyester film with a thickness of 30 µm (Hostaphan RN 302SLK, Mitsubishi Polyester Film GmbH, Wiesbaden, Germany) was used on the bottom of the mold to facilitate the demolding of the frozen sample. At the top of the cold finger and near the brass piece, a resistance heater was installed while the other side of the cold finger was immersed in liquid nitrogen. A thermocouple was positioned in the brass piece to control and indicate the interface temperature between the solution and the brass piece. The solutions were frozen when the temperature indicated by the thermocouple was at −20 °C, −80 °C, and −120 °C. After complete freezing, the samples were placed inside a freezer at the lowest temperature at −20 °C for 72 h for sufficient and faster cross-linking while maintaining the samples frozen. Subsequently, the samples were freeze-dried at −20 °C and 5000 µbar. After freeze-drying, the samples were removed from the freeze-dryer and stored at room temperature. Finally, the samples were pyrolyzed under a flow of high-purity nitrogen (99.999%). The heating rate up to 900 °C was 120 K/h and up to 1000 °C was 30 K/h. The samples dwelled at the maximum temperature for 4 h and were subsequently cooled down to room temperature at a cooling rate of 120 K/min.

Cutting of the screens after pyrolysis was performed in multiple steps, first removing a 1 mm layer from each end and discarding it. When the sample thickness was around 2.6 mm, the screens were polished with sandpaper (SiC) with a grit size of 500 and sonicated for at least 30 min until the thickness of the screens was around 1.5 mm ($L_{n}$, *z*-direction). The height (*x*-direction) and width (*y*-direction) of the screens was 55.7 mm and 17.5 mm, respectively. Testing of the screen with 30 wt% and frozen at −120 °C was not possible due to constant cracking after pyrolysis. Table 1 lists the samples in this study.

### 2.3. Characterization

#### Materials Characterization

To validate the homogeneity of the screen’s pore structure, the pore structure was characterized on the outer sides and in the center of the screen. The morphology of the pores in cross-section and lateral view was analyzed using a scanning electron microscope (SEM, Zeiss EVO 10, Carl Zeiss Microscopy GmbH, Zena, Germany) with specimens sputtered with gold (K550, Emitech, Judges Scientific Plc., Wetzlar, UK). The pore window size distribution and open porosity were obtained using Hg intrusion porosimetry (Pascal 140/440, POROTEC GmbH, Haan, Germany). The average pore window size $2R_{\mathrm{merc}}$ and open porosity *ϕ* were obtained from 3 different positions (two edges, center). To further investigate the mechanical strength of the screens during liquid penetration, we tested the maximum flexural strength of the screens through a 3-point bending test following DIN EN 843-1 and using a 5 kN load cell. The dimensions of the rectangular tested samples were 16 mm in length, ~2 mm in width, and (0.7–1.0) mm in thickness. The samples were placed in the center of a sample holder with a 10 mm distance between the support rollers (diameter of 1.5 mm). The crosshead speed was fixed at 0.1 mm min^−1^ and ten samples for each composition were tested.

### 2.4. Assembling of the SC-LADs

For the permeability tests, bubble point tests, and gas–liquid phase-separation tests, the screens were mounted on a PMMA channel forming the screen liquid acquisition device (SC-LAD). The SC-LAD is a rectangular PMMA channel with a circular outlet at the top, three lateral sides that are solid walls, and a remaining side that is a porous wall that enables phase separation. This side of the channel was covered with the ceramic screen and sealed on the edges with silicone rubber (Elastosil E43, Wacker Chemie AG, Munich, Germany) due to its non-reactivity with the test liquid. The width $W_{\mathrm{SC}}$ and height $H_{\mathrm{SC}}$ of this side of the channel were 52 ± 0.05 mm and 13 ± 0.07 mm, respectively. These data were used to calculate the total screen area $A_{\mathrm{SC}}=W_{\mathrm{SC}}\;H_{\mathrm{SC}}=0.00676$ m^2^. Hydrofluoroether (HFE-7500, 3M™ Novec™ 7500 Engineering Fluid, 3M™, Heilbronn, Germany) was used as the test liquid because it is a completely wetting liquid with regard to the sample and channel material. Application-relevant properties of HFE-7500 such as the density $\rho_{L}$, surface tension *σ*, and dynamic viscosity ${µ}_{L}$ from the manufacturer are listed in Table 2.

### 2.5. Bubble Point Tests

**Figure 2:**
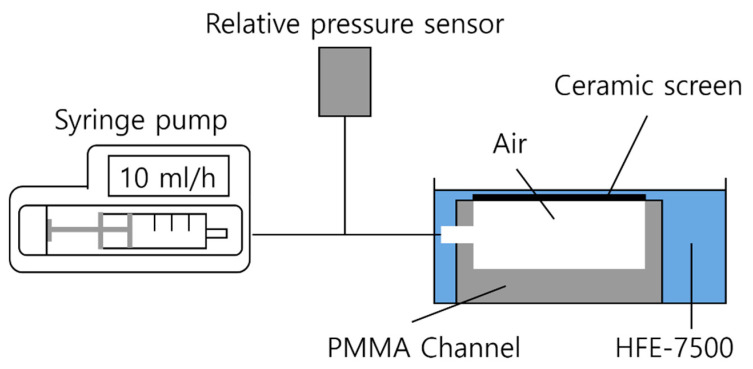
Bubble point testing setup. The pressure inside the channel was increased gradually in a controlled manner by injecting air into the channel with the help of a syringe pump until the bubble breakthrough happened.

In the setup for the bubble point tests, the SC-LAD was placed horizontally in an HFE-7500 liquid bath (Figure 2). The external liquid level was adjusted such that only a thin liquid layer covered the ceramic screen and no test liquid entered the PMMA channel. The outlet of the SC-LAD was connected to a syringe pump which was used to pressurize the channel from the inside with a controlled volumetric flow rate (10 mL h^−1^).

The increment in pressure was measured using a differential pressure sensor from KELLER PR-41/8885 with a pressure range of ±40 mbar. The bubble point pressure is the maximum pressure difference between the liquid and gas phases that the porous screen can sustain. It is a function of the liquid’s surface tension σ, the contact angle θ between the liquid and the solid, and the biggest pore window size, known as the bubble point diameter of the porous screen [3,4], and is given analytically by the following expression.

(1) $$\Delta P_{BP}=\frac{4\sigma \;\cos\;\theta }{D_{\mathrm{BP}}}$$

When the first gas bubble exited from the screen to the liquid bath, the bubble point pressure $\Delta P_{\mathrm{BP}}$ was measured and the bubble point diameter $D_{\mathrm{BP}}$ calculated, which is the size of the biggest available pore window in the porous media. The measurement was repeated three times for each screen.

### 2.6. Gas–Liquid Phase Separation

**Figure 3:**
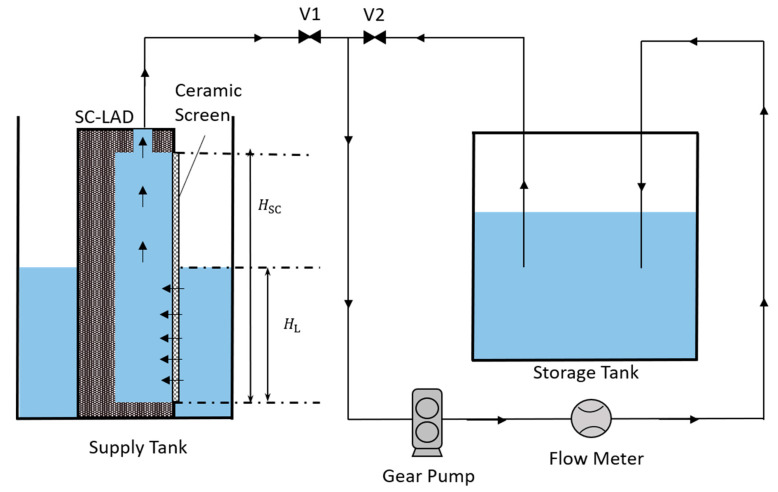
Setup used for testing phase separation. A SC-LAD was placed in a supply tank and the liquid was removed with a constant flow rate with the pump through the SC-LAD to the storage tank.

The phase-separation experiment setup depicted in Figure 3 consisted of the supply tank (containing the SC-LAD, homemade at ZARM institute)), storage tank, gear pump, flow meter, connecting pipes, and operating valves V1 and V2. The gear pump used in the experiment had a gear head (GA-V21, Micropump, Vancouver, DC, USA) along with a variable speed drive (Reglo-Z, ISMATEC-Micropump, Vancouver, DC, USA) to pump the liquid in a loop. The flow meter used for volumetric flow rate measurement was from Kobold LFM. The measuring range of the sensor is 0.08 mL s^−1^ to 4.17 mL s^−1^. The accuracy of the sensor is ±2.5% of the measured value. At the beginning of the experiment, the supply tank was filled with the test liquid, and the SC-LAD was immersed inside the supply tank. With the aid of the gear pump, the SC-LAD was saturated with the liquid, and the gas from all the pipelines was replaced with the liquid. The flow rate of the pump was set to a fixed value and the liquid flow was established in the closed inner loop in which the liquid flowed out of the storage tank and back to the storage tank via the opened valve V2, the gear pump, and the flow meter. When the flow rate became stable, valve V2 was closed and valve V1 was opened simultaneously to start the liquid removal from the supply tank through the SC-LAD. Thus, the liquid flowed from the supply tank to the storage tank via valve V1, the gear pump, and the flow meter.

The liquid enters into the channel through the porous screen and gas ingestion is avoided as long as the overall pressure drop across the porous screen does not exceed the bubble point pressure $\Delta P_{\mathrm{BP}}$. The total pressure drop $\Delta P_{total}$ in the LAD system for a steady state flow in a 1$g_{E}$ environment (normal gravitational acceleration of earth) comprises different pressure drop terms and can be expressed as their summation [6].

(2) $$\Delta P_{total\;}=\Delta P_{\mathrm{HS}}+\Delta P_{\mathrm{FTS}}+\Delta P_{\mathrm{FR}}+\Delta P_{\mathrm{DY}}$$

$\Delta P_{\mathrm{HS}}$ is the hydrostatic pressure drop across the channel, which depends on the height of the screen exposed to the gaseous phase $\left(H_{\mathrm{SC}}-H_{L}\right).$ The pressure drop due to the flow of the liquid across the porous screen is represented by the term $\Delta P_{\mathrm{FTS}}$. The frictional pressure drop due to the friction between the LAD wall and the liquid is represented by $\Delta P_{\mathrm{FR}}$. The last term $\Delta P_{\mathrm{DY}}$ accounts for the dynamic pressure loss due to the inflow of liquid into the channel. In the present phase-separation experiment, based on the geometry and dimension of the LAD, it was found that $\Delta P_{\mathrm{HS}}$ and $\Delta P_{\mathrm{FTS}}$ are the major contributing terms to the $\Delta P_{total}$. Therefore, the contribution of other terms is neglected and Equation (3) is used.

(3) $$\Delta P_{total\;}=\Delta P_{\mathrm{HS}}+\Delta P_{\mathrm{FTS}}$$

To prevent gas ingestion inside the channel, the following condition should be met:

(4) $$\;\Delta P_{\mathrm{BP}}\geq \;\Delta P_{total\;}$$

A further explanation of the calculation of each pressure is given in the next section, where the theoretical background is discussed in detail. For the experimental results, each SC-LAD was tested 3 times. All experiments were recorded using an optical system that consists of a CMOS camera (from Imaging source) and a Stemmer Imaging LED light panel. When gas ingestion started, the height of the screen exposed to the liquid $H_{L}\;$(also referred to as breakthrough height further in the text) was noted (Figure 3 and Figure S1) and multiplied by the width of the channel $W_{\mathrm{SC}}$ to calculate the area exposed to the liquid $A_{\mathrm{SC}}$ (effective screen area).

(5) $$A_{\mathrm{SC}}=H_{L}W_{\mathrm{SC}}$$

$A_{\mathrm{SC}}$ denotes the area of the ceramic screen through which liquid is entering the SC-LAD from the supply tank. While the removal volumetric flow rate$\;\dot{V_{L}}$ is constant, the decrease in the liquid in the supply tank decreases the area of the screen through which liquid enters the channel. This leads to an increase in $\Delta P_{\mathrm{FTS}}$ and $\Delta P_{\mathrm{HS}}$. Therefore, when their sum becomes equal to $\Delta P_{\mathrm{BP}}$, the screen starts ingesting gas bubbles into the channel. This point of gas ingestion is referred to as the critical point in the presented work. For each removal volumetric flow rate$\;\dot{V_{L}}$, the sum of $\Delta P_{\mathrm{HS}}$ and $\Delta P_{\mathrm{FTS}}$ will become equal to $\Delta P_{\mathrm{BP}}\;$at a critical value of $H_{L}$ and below this value, bubbles will be ingested into the channel. The experiment was performed for different flow rates in the range of 0.5 mL s^−1^ to 4 mL s^−1^ at room temperature.

### 2.7. Theoretical Approach

In our experimental setup, gas ingestion through the ceramic screen during the liquid removal from the SC-LAD can be avoided as long as the total pressure drop $\Delta P_{total}$ in the LAD system is smaller than the bubble point (Equations (3) and (4)). Under these experimental conditions, $\Delta P_{\mathrm{HS}}$ and $\Delta P_{\mathrm{FTS}}$ can be considered the most important terms contributing to the total pressure drop across the screen. Thus, as long as the bubble point pressure is higher than the sum of the hydrostatic pressure $\Delta P_{\mathrm{HS}}$ and the flow-through-screen pressure $\Delta P_{\mathrm{FTS}}$, no gas breakthrough should occur.

(6) $$\Delta P_{\mathrm{BP}}\geq \;\Delta P_{\mathrm{HS}}+\Delta P_{\mathrm{FTS}}\;$$

$\Delta P_{\mathrm{BP}}$ is given by Equation (1). $\Delta P_{\mathrm{HS}}$ is given by:

(7) $$\Delta P_{\mathrm{HS}}=\rho_{L}g_{E}\left(H_{\mathrm{SC}}-H_{L}\right)$$

$g_{E}\;$is the gravitational acceleration on the Earth.

$\Delta P_{\mathrm{FTS}}$ is calculated using Darcy’s law as given in Equation (8). It governs the flow through any porous media under the application of a certain pressure gradient or vice versa. It assumes that the viscous pressure loss through a specimen is linearly dependent on the flow rate of the fluid [36]. The experimental method assumes the validity of the Darcy equation (Equation (8)) in the scope of our setup, where $\Delta P_{\mathrm{FTS}}$ is the flow-through-screen pressure drop across the screen’s thickness $L_{n}$, having a permeability $K_{D}$ with an applied superficial velocity of $v_{S}$. The superficial velocity $v_{S}$ is defined as the volumetric flow rate $\dot{V_{L}}$ divided by the effective screen area $A_{\mathrm{SC}}$ (given by Equation (5)). The superficial velocity $v_{S}$ is assumed to approach the test sample with a uniform magnitude.

(8) $$\Delta P_{\mathrm{FTS}}=\frac{{µ}_{L}L_{n}}{K_{D}}v_{S}=\frac{{µ}_{L}L_{n}}{K_{D}}\frac{\dot{V_{L}}}{A_{\mathrm{SC}}}$$

At the critical point, when $\Delta P_{\mathrm{BP}}$ and $\Delta P_{total\;}$ balance each other, bubble breakthrough occurs and the SC-LAD will no longer be able to separate the liquid and gaseous phases. Thus, Equation (6) can be written as Equation (9) for the pressure balance at the critical point.

(9) $$\Delta P_{\mathrm{BP}}=\Delta P_{\mathrm{HS}}+\Delta P_{\mathrm{FTS}}$$

$\Delta P_{\mathrm{HS}}$ can be calculated with the help of the breakthrough height $H_{L}$ from the phase-separation experiment using Equation (7) and $\Delta P_{\mathrm{BP}}$ is known from the bubble point test. The flow-through-screen pressure drop $\Delta P_{\mathrm{FTS}}$ can be calculated from Equation (9). Consequently, the permeability $K_{D}$ can be obtained using Equation (8).

Thus, considering the properties of the ceramic screen, the bubble point of the screen, the properties of the test liquid, the breakthrough height $H_{L}$ from the phase-separation experiment, and the corresponding volumetric flow rate $\dot{V_{L}}$ as known quantities, Equations (7) and (8) can be substituted in Equation (9) to obtain the permeability $K_{D}$ for each set of $\dot{V_{L}}$ and $H_{L}$.

(10) $$K_{D}=\frac{{µ}_{L}L_{n}{}_{\;}}{\left(\Delta P_{\mathrm{BP}}-\rho_{L}g_{E}\left(H_{\mathrm{SC}}-H_{L}\right)\right)}\frac{\dot{V_{L}}}{H_{L}W_{\mathrm{SC}}}$$

We will now consider two special cases.

(a) No-flow case:

If the SC-LAD is completely filled with liquid and removed from the liquid pool, it will hold the liquid as long as

(11) $$\Delta P_{\mathrm{BP}}\geq \;\Delta P_{\mathrm{HS}\;}$$

(12) $$\Delta P_{\mathrm{BP}}\geq \;\rho_{L}g_{E}H_{\mathrm{SC}}$$

An equilibrium height $h_{\mathrm{EH}}$ can be defined where the bubble point pressure $\Delta P_{\mathrm{BP}}$ is balanced by the hydrostatic pressure $\Delta P_{\mathrm{HS}\;}$.

(13) $$h_{\mathrm{EH}}=\frac{\Delta P_{\mathrm{BP}}}{\rho_{L}g_{E}}$$

The SC-LAD can only hold the liquid under static conditions when $H_{\mathrm{SC}}\;\leq \;h_{\mathrm{EH}}$.

**Figure 4:**
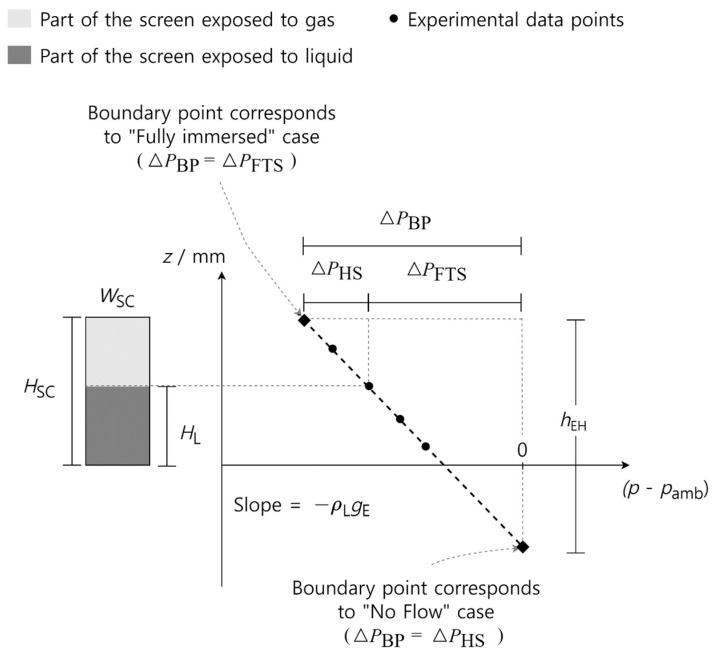
Schematic diagram of the graphical representation of results from the gas−liquid phase-separation experiment.

If we plot the *z*-position versus the pressure difference $\left(p-p_{\mathrm{amb}}\right)$, as shown in Figure 4, a slope can be defined as

(14) $$\frac{dp}{dz}=-\rho_{L}g_{E}$$

The negative sign denotes that the pressure decreases in the direction of positive *z*, and $p_{\mathrm{amb}}$ is the ambient pressure.

(b) Fully immersed case:

If the SC-LAD is fully immersed in the liquid pool, the effect of the hydrostatic pressure across the screen is zero. Hence, Equation (6) will give a boundary point where

(15) $$\Delta P_{\mathrm{BP}}\geq \;\Delta P_{\mathrm{FTS}\;}$$

A maximum flow rate can be computed from Equation (8) as follows:

(16) $$({\dot{V_{L})}}_{\max}=\frac{K_{D}}{{µ}_{L}}\frac{A_{\mathrm{SC}}}{L_{n}}\Delta P_{\mathrm{BP}}$$

## 3. Results and Discussion

This section starts with a description and discussion of the pore window size distribution, bubble point diameter, porosity, and pore morphology of the created screens. Subsequently, the influence of the pore structure on the results of permeability and phase-separation tests using the SC-LAD will be discussed.

### 3.1. Pore Morphology

**Figure 5:**
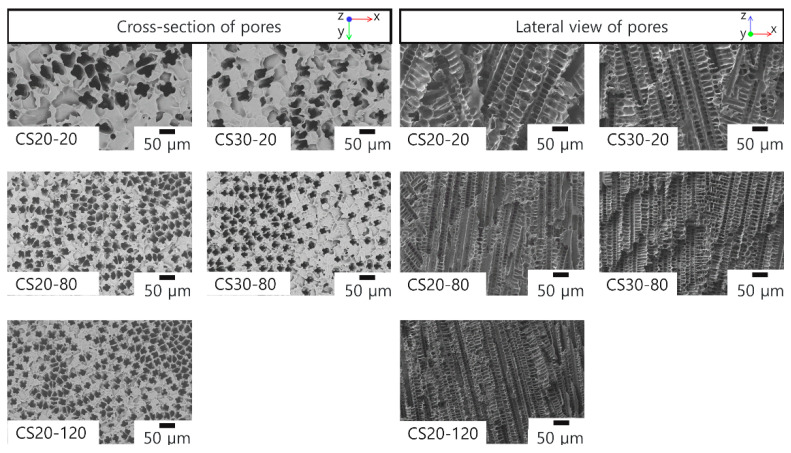
SEM pictures of pore morphology for the center position of the samples. Images of cross-section and lateral view with pores perpendicular and parallel to the freezing direction, respectively.

The pore morphology was analyzed in the direction perpendicular (cross-section of pores) and parallel (lateral view of pores) to the freezing direction. The SEM images presented in Figure 5 are from the center part of the screen and show no difference compared to the edges of the samples.

The SEM images show the formation of a dendritic pore morphology which typically occurs when cyclohexane is used as a solvent during freeze-casting [33,37]. The lateral view of the pores shows an aligned pore structure with primary dendritic arms (bigger pore size) and secondary dendritic arms (smaller pore size) parallel and perpendicular to the mass transport direction, respectively. The SEM images from the cross-section show the exclusive formation of primary dendritic pore windows and the absence of macro defects such as cracks or abnormally large pores. Furthermore, Figure 5 shows that the size of the frozen solvent crystals, and thus the pore size, decreases as the freezing temperature decreases (i.e., the degree of supercooling increases) [15]. Higher-magnification images of SEM are placed in the supplementary data, in Figure S4.

### 3.2. Pore Window Size Distribution and Bubble Point Diameter

**Figure 6:**
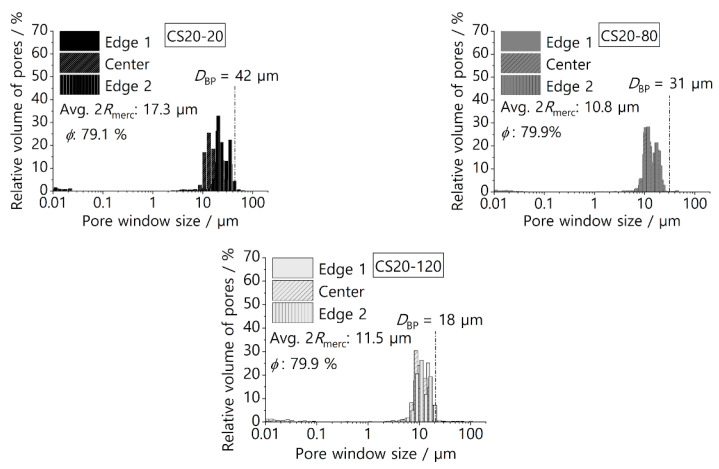
Hg intrusion porosimetry diagram for samples with a solid loading of 20 wt% for different freezing temperatures. Dashed lines indicate the biggest pore window size calculated based on bubble point pressure $D_{\mathrm{BP}}$.

**Figure 7:**
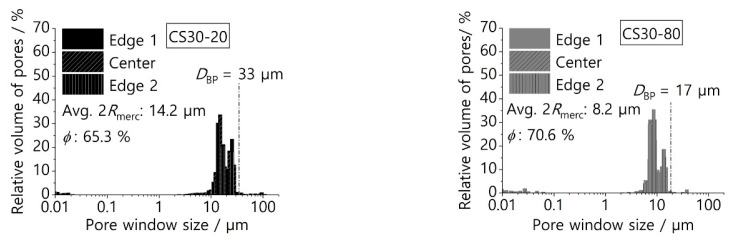
Hg intrusion porosimetry diagram for samples with 30 wt% of solid loading for different temperatures of freezing. Dashed lines indicate the biggest pore window size calculated based on the bubble point pressure $D_{\mathrm{BP}}$.

Figure 6 and Figure 7 show the pore window size distribution with average values of pore window size and open porosity for samples with 20 wt% and 30 wt% and frozen at different temperatures. Samples with a higher solid loading (40 wt%) were created in preliminary studies, but they possessed lower porosity (around 54%), which would create an excessive pressure drop during application. Samples with a lower solid loading (10 wt%) possess a higher open porosity but are mechanically unstable.

Overall, the average pore window size varies between 8.2 µm and 17.3 µm depending on the freezing temperature and solid loading, and the pore sizes can be found in the range between 6 µm and 43 µm. Within the same sample, an extensive overlap of pore size distribution from the edges and the center of the screen can be seen, which confirms (quantitatively) the homogeneity of the screens. The quantitative results shown in Figure 6 and Figure 7 confirm the qualitative observations in Figure 5 regarding the decrease in pore window size with the decrease in freezing temperature. The average open porosity varies between 65.3% and 79% and is mainly influenced by the solid loading. This correlation is documented in the literature and applies to samples produced by freeze-casting of either oxidic ceramic particles or preceramic polymers [38]. Figure 6 shows that screens with a solid loading of 20 wt% have a wider and more homogenous pore size distribution. Thus, a clear distinction between primary and secondary dendritic arms is not viable for screens with 20 wt%. In contrast, the screens with 30 wt% show a bimodal pore window size distribution, and a distinction between the secondary and primary dendritic arms is possible here (Figure 7). The ratio between the pore window size of primary and secondary dendritic arms are 1.68 and 1.75 for screens frozen at −20 °C and −80 °C, respectively. These values are close to the ratio of 2 already reported in the literature for aligned dendritic pore structures created from freeze-casting of preceramic polymers [39,40]. For samples with a higher solid loading, although the primary dendritic arms still have a greater influence on mass transfer within the pore structure due to their orientation in the direction of flow, the numerous secondary dendritic arms that grow from a primary dendrite often have a much larger pore volume [39]. These secondary pores are often not interconnected [41] and are perpendicular to the mass flow, thus representing an additional path for liquid flow and slowing it down during the phase separation. Moreover, the values of the bubble point diameter $D_{\mathrm{BP}}$ were calculated using Equation (1) and the measured bubble point pressure. These $D_{\mathrm{BP}}$ values are shown in Figure 6 and Figure 7 for comparison and for each screen, the value of $D_{\mathrm{BP}}$ falls close to the biggest pore window sizes measured with Hg intrusion. This cross-check of the biggest pore diameter was important for the LAD development, as it showed that there were no major defects or leaking joints (e.g., cracks or pores bigger than 50 µm). In addition, the screens were tested in three-point bending tests, as space applications generally require good mechanical stabilities and thus corresponding long-term stability of the materials. Figure S2 shows flexural strength values from 12.41 MPa to 16.76 MPa with the open porosity having the greatest influence on the mechanical strength. Due to these good mechanical stabilities, the screens broke neither during the assembly of the SC-LADs nor during the gas-liquid phase separation. To confirm the mechanical stability of the screens during phase separation and that the liquid removed through the SC-LAD was particle-free, 10 mL of the extracted liquid was collected after testing every applied removal volumetric flow rate. This procedure was performed on sample CS20-20, as this screen showed the biggest pore window sizes and higher open porosity, and therefore, should possess the lowest mechanical stability. The collected liquid was filtered using a paper filter and Figure S3 shows SEM images of filter paper before filtering the liquid and after gas–liquid phase separation. Results from the pore structure characterization, sample dimensions, and permeability values are summarized in Table 3 below. Permeability values are discussed in the next section.

### 3.3. Phase Separation and Permeability

**Figure 8:**
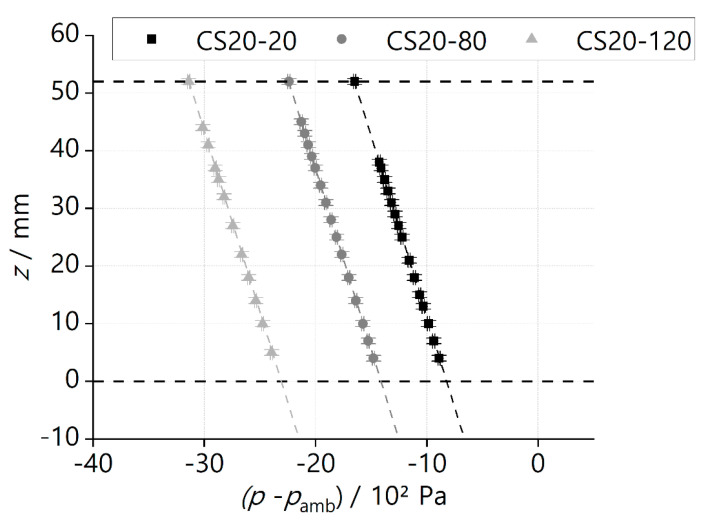
Experimental data (symbols) and calculated hydrostatic head (dotted lines, Equation (14)) from phase−separation experiment at different flow rates for samples with 20 wt% of solid loading. Details corresponding to each data point are given in Table 4, Table 5 and Table 6. The vertical error bar for height measurement *z* is ±0.5 mm and the horizontal error bar for pressure difference ($p-p_{\mathrm{amb}}$) is ±0.1 hPa.

**Figure 9:**
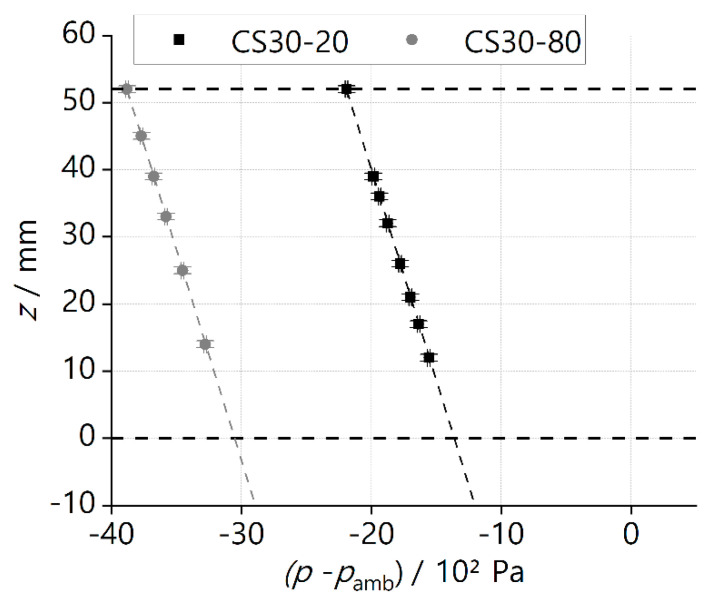
Experimental data (symbols) and calculated hydrostatic head (dotted lines, Equation (14)) from phase−separation experiment at different flow rates for samples with 30 wt% of solid loading. Details corresponding to each data point are given in Table 7 and Table 8. The vertical error bar for height measurement *z* is ±0.5 mm and the horizontal error bar for pressure difference ($p-p_{\mathrm{amb}}$) is ±0.1 hPa.

The phase-separation experiments were performed to test and compare the performance of different ceramic screens. The liquid was withdrawn from the supply tank through the SC-LAD with a constant volumetric flow rate $\dot{V_{L}}$. At the point of bubble point breakthrough, the height of the screen exposed to the liquid was noted as the breakthrough height $H_{L}$. The experiment parameter and results obtained from the phase-separation experiment performed for different ceramic samples are presented in Table 4, Table 5, Table 6, Table 7 and Table 8. From the recorded images of the phase-separation experiment, the value of $H_{L}$ corresponding to the first ingested bubble was obtained. At this critical point, $\Delta P_{\mathrm{HS}}$, $\Delta P_{\mathrm{FTS}}$, and $K_{D}$ are calculated using Equations (7), (9), and (10), respectively. Equations (13) and (16) are used to calculate $h_{\mathrm{EH}}$ and $({\dot{V_{L})}}_{\max}$ for each sample. Figure 8 and Figure 9 represent the phase-separation data graphically where the breakthrough height $H_{L}$ obtained for the different flow rate $\dot{V_{L}}$ is plotted against the pressure difference $p-p_{\mathrm{amb}}$. $p_{\mathrm{amb}}$ is the ambient pressure.

A schematic diagram explaining the graphical representations of the data can be found in Figure 4. The dotted line represents the theoretical linear curve of the hydrostatic head. The theoretical endpoints are calculated analytically from the ‘no-flow’ and ‘fully immersed’ cases. The top boundary point corresponds to the ‘fully immersed’ case, where the whole sample screen is exposed to the liquid with a maximum removal flow rate such that $\Delta P_{\mathrm{BP}}$ is balanced by $\Delta P_{\mathrm{FTS}}$. At this point, the x coordinate shows the $\Delta P_{\mathrm{BP}}$ of the sample. The bottom boundary point (not shown in Figure 8 and Figure 9) corresponds to the ‘no-flow’ case where $\Delta P_{\mathrm{BP}}$ is balanced by $\Delta P_{\mathrm{HS}}$ corresponding to an imaginary screen height of $h_{\mathrm{EH}}$. Figure 8 and Figure 9 show the experimental data of the ceramic screens with 20 wt% solid loading and 30 wt% solid loading, respectively. All SC-LADs showed an increase in $H_{L}$ at the critical point (i.e., when bubble ingestion starts) with an increase in the applied removal volumetric flow rate $\dot{V_{L}}$. This correlation implies that the flow-through-screen pressure drop $\Delta P_{\mathrm{FTS}}$ increases with the applied removal volumetric flow rate, and therefore the sum of $\Delta P_{\mathrm{FTS}}$ and $\Delta P_{\mathrm{HS}}$ is balanced by the $\Delta P_{\mathrm{BP}}$ of the ceramic screen at higher values of $H_{L}$. Thus, the larger the removal flow rate $\dot{V_{L}}$, the larger the critical effective screen area $A_{\mathrm{SC}}$. SC-LADs prepared with screens with higher open porosity (i.e., 20 wt%) were able to perform phase separation during the experiment up to a removal flow rate of 4 mL s^−1^ without bubble ingestion, whereas SC-LADs prepared with screens with less open porosity (i.e., 30 wt%) were limited to a flow rate of 2 mL s^−1^. This can also be seen by comparing $({\dot{V_{L})}}_{\max}$ for each sample screen. The maximum allowable removal flow rate $({\dot{V_{L})}}_{\max}$ is higher for SC-LADs with 20 wt% as compared to 30 wt%. A higher open porosity represents more pathways for the liquid to flow through the screen and therefore, less flow-through-screen pressure drop $\Delta P_{\mathrm{FTS}}$ during gas–liquid phase separation.

When comparing SC-LADs with ceramic screens with the same open porosity, the smaller the pore window sizes of the screen, the more sensitive the SC-LAD is to an increase in the applied volumetric flow. This observation indicates a correlation between the decrease in pore window size with the increase in the flow-through-screen pressure drop $\Delta P_{\mathrm{FTS}}$. Upon analyzing the values of $K_{D}$ reported for each sample in Table 4, Table 5, Table 6, Table 7 and Table 8, it can be observed that it converges to a relatively constant value at a higher effective screen area $A_{\mathrm{SC}}$. It seems that when $A_{\mathrm{SC}}$ reaches higher values such as more than half of the total screen area, the value of $K_{D}$ starts to converge. The average of this converged value of $K_{D}$ can be considered as the average permeability of the whole sample, which is reported for each sample in Table 3. Interestingly, the value of $K_{D}$ is almost consistent for all data points for SC-LADs with a lower open porosity (~65%) as their pore size distribution is narrower as compared to SC-LADs with a higher open porosity (~79%). In this work, the manufacturing method used and the resulting ceramic screens provide a tool kit for creating screens for phase separation in a wide volume range. Aside from the application on which this work is focused (gas–liquid phase separation), the screens could also be used for other separation techniques that require a pore window size of the primary dendritic arms between 10 µm and 40 µm. By adding fillers to the composition of the screen, the surface roughness of the pore wall can also be adjusted so that the particles adhere or are detached optimally during, e.g., deep-bed filtration [8]. Nevertheless, the addition of filler must also be controlled to avoid a significant disturbance in crystal formation during freezing, compromising the formation of a stable dendritic pore morphology.

## 4. Conclusions

SiOC screens with different pore window size distributions and open porosities were prepared through solution-based freeze-casting of preceramic polymer and employed successfully as a porous screen in a SC-LAD for a phase-separation application. The ceramic screens show a homogenous pore structure along their cross-section. The pore window sizes ranged between 6 µm to 43 µm depending on the freezing temperature during freeze-casting of the polymer solution. When producing a screen with higher solvent contents (template media), the solid loading is reduced from 30 wt% to 20 wt%, which increases the resulting open porosity from 65% to 79%. In contrast, preparing a screen from a less diluted solution (i.e., solid loading 30 wt%) results in a distinct bimodal size distribution of the pore windows. Permeability values calculated from the phase-separation experiment range from 1.4 × 10^−12^ m^2^ to 9.1 × 10^−12^ m^2^, decreasing overall as freezing temperature is lowered or solid loading is increased during screen manufacture. The calculated bubble point diameter follows the same trend and a cross-check with the pore window size distributions proves that all produced screens are crack-free and can be applied as SC-LADs in gas–liquid phase separation as well as in a variety of different applications where phase separation is a requirement. The SC-LADs can supply different amounts of gas-free liquid depending on the applied volumetric flow and the pore structure of the SC-LADs. In our phase-separation experiment setup, we tested different groups of SC-LADs with removal flow rates from 0.5 mL s^−1^ to 4 mL s^−1^. The SC-LADs with lower open porosity (~65%) were able to supply gas-free liquid up to a removal flow rate of 2 mL s^−1^, whereas almost all the SC-LADs with a higher open porosity (~79%) were able to supply liquid up to 4 mL s^−1^. The same behavior can be confirmed by comparing the $({\dot{V_{L})}}_{\max}$ of each sample. This indicates that $\Delta P_{\mathrm{FTS}}$ was higher for SC-LADs with a lower open porosity as compared to SC-LADs with a higher open porosity. Additionally, SC-LADs with smaller pore window sizes within the same porosity group also showed higher pressure drops and increased sensitivity of the phase-separation performance to the applied volumetric flow rates. Upon analyzing the calculated $K_{D}$ values corresponding to different flow rates and respective breakthrough heights, it was observed that the value converges to a relatively constant value at higher effective areas of the screen. Interestingly, for the SC-LADs with lower open porosity (~65%), the value of $K_{D}$ remained more or less constant at all the data points as the pore size distribution in this group is narrower in comparison to SC-LADs with high open porosity (~79%). Due to their chemical stability and low thermal conductivity, SiOC screens may be a promising option for applications with harsh chemical conditions and a broader range of working temperatures (e.g., high temperature or cryogenic temperatures). Further extensions of this study include using asymmetric SiOC screens that have an additional thin top layer with a smaller pore size (<3 µm). This might require an additional process step, but it could increase the bubble point pressure while maintaining a low pressure drop across the screen. As an outlook, testing the SiOC screens using cryogenic test liquids and/or in microgravity conditions could better simulate the final application conditions of SC-LADs in propellant-management devices. 

## Figures and Tables

**Table 1:** Denotation, solid loading, and freezing conditions.

Denotation	Solid Loading/wt%	Freezing Temperature/°C
CS20-20	20	−20
CS20-80	20	−80
CS20-120	20	−120
CS30-20	30	−20
CS30-80	30	−80

**Table 2:** Characteristics of liquid HFE-7500 at 25°C.

Property	Value	Unit
$\rho_{L}$	1614	kg m^−3^
σ	16.2 × 10^−3^	N m^−1^
$\mu_{L}$	1.24 × 10^−3^	Pa s
$\nu_{L}$	0.77 × 10^−6^	m^2^ s^−1^

**Table 3:** Geometric and macroscopic parameters of the ceramic screens.

Denotation	$2\;R_{merc}/10^{-6}\;m$	*Φ*	$\Delta P_{BP}/10^{-3}\;\mathrm{bar}$	$K_{D}/10^{-12}\;m^{2}$	$L_{n}/10^{-3}\;m$
CS20-20	17.3 ± 3.7	0.791 ± 0.026	16.5 ± 0.14	9.1 ± 0.6	1.31 ± 0.02
CS20-80	10.7 ± 0.9	0.799 ± 0.018	22.4 ± 0.26	5.1 ± 0.6	1.28 ± 0.11
CS20-120	11.5 ± 3.1	0.799 ± 0.015	31.4 ± 0.19	3.6 ± 0.6	1.67 ± 0.01
CS30-20	14.2 ± 0.7	0.653 ± 0.038	21.9 ± 0.05	4.4 ± 0.6	1.76 ± 0.11
CS30-80	8.2 ± 0.8	0.706 ± 0.049	38.8 ± 0.05	1.4 ± 0.6	1.65 ± 0.01

**Table 4:** Phase-separation experiment parameters and results for sample CS20-20 with $\Delta P_{\mathrm{BP}}\;$ = 16.5 hPa, $h_{\mathrm{EH}}$ = 104.2 mm, and $({\dot{V_{L})}}_{\max}$ = 6.25 mL s^−1^.

CS20-20				
$\dot{V_{L}}/\mathrm{mL}\;s^{-1}$	$H_{L}/\mathrm{mm}$	$\Delta P_{HS}/\mathrm{hPa}$	$\Delta P_{FTS}/\mathrm{hPa}$	$K_{D}/10^{-12}\;m^{2}$
0.50	4	7.6	8.9	17.5
0.75	7	7.1	9.4	14.3
1.00	10	6.7	9.8	12.7
1.25	13	6.2	10.3	11.6
1.50	15	5.9	10.6	11.7
1.75	18	5.4	11.1	10.9
2.00	21	4.9	11.6	10.3
2.25	25	4.3	12.2	9.2
2.50	27	4.0	12.5	9.2
2.75	29	3.6	12.9	9.2
3.00	31	3.3	13.2	9.2
3.25	33	3.0	13.5	9.1
3.50	35	2.7	13.8	9.0
3.75	37	2.4	14.1	9.0
4.00	38	2.2	14.3	9.2

**Table 5:** Phase-separation experiment parameters and results for sample CS20-80 with $\;\Delta P_{\mathrm{BP}}\;$ = 22.4 hPa, $h_{\mathrm{EH}}$ = 141.5 mm, and $({\dot{V_{L})}}_{\max}$ = 4.87 mL s^−1^.

CS20-80				
$\dot{V_{L}}/\mathrm{mL}\;s^{-1}$	$H_{L}/\mathrm{mm}$	$\Delta P_{HS}/\mathrm{hPa}$	$\Delta P_{FTS}/\mathrm{hPa}$	$K_{D}/10^{-12}\;m^{2}$
0.50	4	7.6	14.8	10.3
0.75	7	7.1	15.3	8.6
1.00	10	6.7	15.7	7.8
1.25	14	6.0	16.4	6.7
1.50	18	5.4	17.0	6.0
1.75	22	4.8	17.6	5.5
2.00	25	4.3	18.1	5.4
2.25	28	3.8	18.6	5.3
2.50	31	3.3	19.1	5.2
2.75	34	2.9	19.5	5.1
3.00	37	2.4	20.0	4.9
3.25	39	2.1	20.3	5.0
3.50	41	1.7	20.7	5.0
3.75	43	1.4	21.0	5.1
4.00	45	1.1	21.3	5.1

**Table 6:** Phase-separation experiment parameters and results for sample CS20-120 with $\Delta P_{\mathrm{BP}\;}$ = 31.4 hPa, $h_{\mathrm{EH}}$ = 198.3 mm, and $({\dot{V_{L})}}_{\max}$ = 3.49 mL s^−1^.

CS20-120				
$\dot{V_{L}}/\mathrm{mL}\;s^{-1}$	$H_{L}/\mathrm{mm}$	$\Delta P_{HS}/\mathrm{hPa}$	$\Delta P_{FTS}/\mathrm{hPa}$	$K_{D}/10^{-12}\;m^{2}$
0.50	5	7.4	24.0	6.6
0.75	10	6.7	24.7	4.8
1.00	14	6.0	25.4	4.5
1.25	18	5.4	26.0	4.3
1.50	22	4.8	26.6	4.1
1.75	27	4.0	27.4	3.8
2.00	32	3.2	28.2	3.5
2.25	35	2.7	28.7	3.6
2.50	37	2.4	29.0	3.7
2.75	41	1.7	29.7	3.6
3.00	44	1.3	30.1	3.6

**Table 7:** Phase-separation experiment parameters and results for sample CS30-20 with $\Delta P_{\mathrm{BP}}\;$ = 21.9 hPa, $h_{\mathrm{EH}}$ = 138.3 mm, and $({\dot{V_{L})}}_{\max}$ = 2.98 mL s^−1^.

CS30-20				
$\dot{V_{L}}/\mathrm{mL}\;s^{-1}$	$H_{L}/\mathrm{mm}$	$\Delta P_{HS}/\mathrm{hPa}$	$\Delta P_{FTS}/\mathrm{hPa}$	$K_{D}/10^{-12}\;m^{2}$
0.50	12	6.3	15.6	4.5
0.75	17	5.5	16.4	4.5
1.00	21	4.9	17.0	4.7
1.25	26	4.1	17.8	4.5
1.50	32	3.2	18.7	4.2
1.75	36	2.5	19.4	4.2
2.00	39	2.1	19.8	4.3

**Table 8:** Phase-separation experiment parameters and results for sample CS30-80 with $\Delta P_{\mathrm{BP}}\;$ = 38.8 hPa, $h_{\mathrm{EH}}$ = 245 mm and $({\dot{V_{L})}}_{\max}$ = 1.79 mL s^−1^.

CS30-80				
$\dot{V_{L}}/\mathrm{mL}\;s^{-1}$	$H_{L}/\mathrm{mm}$	$\Delta P_{HS}/\mathrm{hPa}$	$\Delta P_{FTS}/\mathrm{hPa}$	$K_{D}/10^{-12}\;m^{2}$
0.50	14	6.0	32.8	1.7
0.75	25	4.3	34.5	1.4
1.00	33	3.0	35.8	1.3
1.25	39	2.1	36.7	1.4
1.50	45	1.1	37.7	1.4

## Data Availability

Not applicable.

## Supplementary Materials

The following supporting information can be downloaded at: https://www.mdpi.com/article/10.3390/ma16031063/s1, Figure S1: Vertically oriented SC-LAD schematic with partial filled supply tank in $g_{E}$; Figure S2: Values of flexural strength from 3-point bending tests, Figure S3: SEM images of paper filter before and after filtering the liquid in the outlet of the gas-liquid phase separation, Figure S4: SEM pictures with higher resolution of pore structures.

## Nomenclature

$2\;R_{merc}$	Average pore window size	µm
$A_{\mathrm{SC}}$	Effective area of screen exposed to liquid	mm^2^
$D_{\mathrm{BP}}$	Bubble point diameter	m
$g_{E}$	Gravitational acceleration on earth	9.81 m s^−2^
$h_{\mathrm{EH}}$	Equilibrium height of the ceramic screen	mm
$H_{L}$	Height of the ceramic screen immersed in the liquid	mm
$H_{\mathrm{SC}}$	Total height of the ceramic screen	mm
$K_{D}$	Darcian permeability of the ceramic screen	m^2^
	Thickness of the screen in the normal direction	mm
M	Molar mass	g mol^−1^
p	Pressure	Pa
$p_{\mathrm{amb}}$	Ambient pressure	Pa
$v_{S}$	Superficial or filter velocity	m s^−1^
$\dot{V_{L}}$	Volumetric flow rate	ml s^−1^
$({\dot{V_{L})}}_{\max}$	Maximum volumetric flow rate allowed	ml s^−1^
$W_{\mathrm{SC}}$	Width of the ceramic screen exposed to liquid transport	m
$\Delta P_{total\;}$	Total pressure drop in the LAD system	Pa
$\Delta P_{\mathrm{BP}}$	Bubble point pressure	Pa
$\Delta P_{\mathrm{FTS}}$	Flow-through-screen pressure drop	Pa
$\Delta P_{\mathrm{HS}}$	Hydrostatic pressure difference	Pa
$\Delta P_{\mathrm{FR}}$	Frictional pressure drop	Pa
$\Delta P_{\mathrm{DY}}$	Dynamic pressure loss	Pa
Δ	Difference	-
θ	Contact angle	°
${µ}_{L}$	Dynamic viscosity of the liquid	Pa s
$\rho_{L}$	Liquid density	kg m^−3^
$\nu_{L}$	Kinematic viscosity of the liquid	m^2^ s^−1^
σ	Surface tension	N m^−1^
wt	Solid loading, mass fraction	%
*ϕ*	Porosity	%
