# Supplementary material for: SiOC Screens with Aligned and Adjustable Pore Structure for Screen Channel Liquid Acquisition Device
Source: Materials (Basel). 2023 Jan 25;16(3):1063. doi: 10.3390/ma16031063 (PMC9920289; doi:10.3390/ma16031063)
Supplement: Supplementary file 1 [file materials-16-01063-s001.zip › materials-2143994-supplementary.pdf]

Supplementary material

Pedro Henrique da Rosa Braun[1†], Prithvi Shukla [2†], Kurosch Rezwan[1, 3], Michael Dreyer [2], Michaela Wilhelm[1*]

[1] University of Bremen, Advanced Ceramics, Am Biologischen Garten 2, IW3, Germany

[2] University of Bremen, Faculty of Production Engineering - Mechanical and Process Engineering, Department of Fluid mechanics, ZARM, Am Fallturm 2, Bremen, Germany

[3] MAPEX—Center for Materials and Processes, University of Bremen, Am Fallturm 1, Bremen, Germany

† Equal authorship * Corresponding author. Tel.: +49 421 218 64944; E-mail address: mwilhelm@unibremen.de.

List of Abbreviation

APTES	(3-aminopropyl) triethoxysilane
CH	Cyclohexane
CS	Ceramic screen
LAD	Liquid acquisition device
PMD	Propellent management device
SC-LAD	Screen channel liquid acquisition device

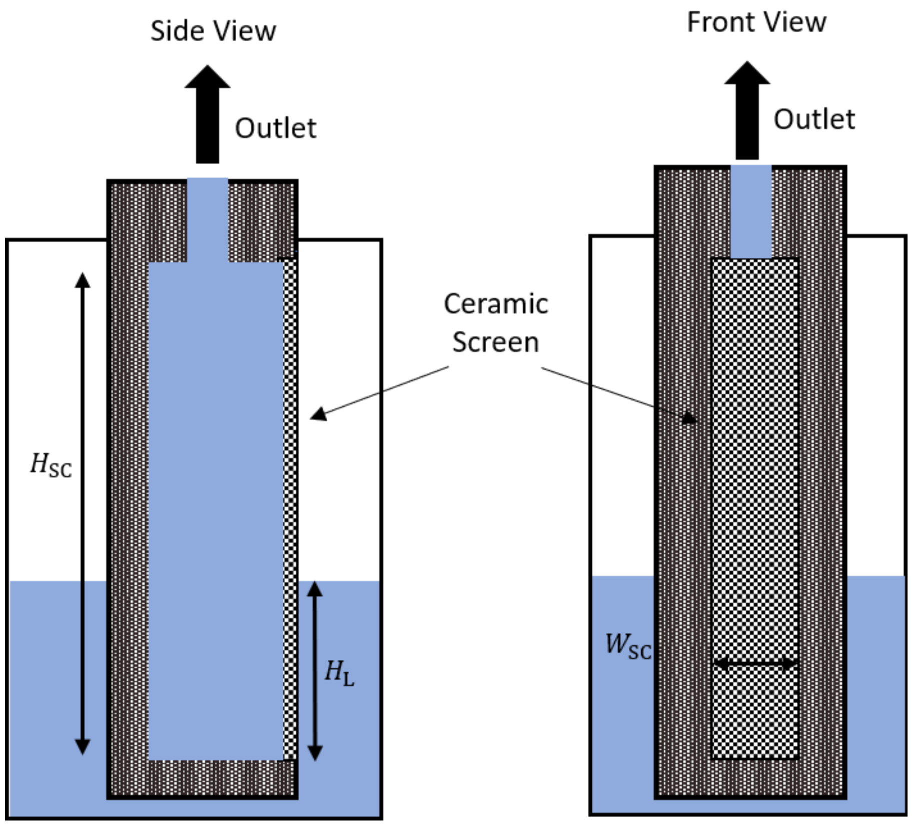

Figure S1 Vertically oriented SC-LAD schematic with partial filled supply tank in g_E

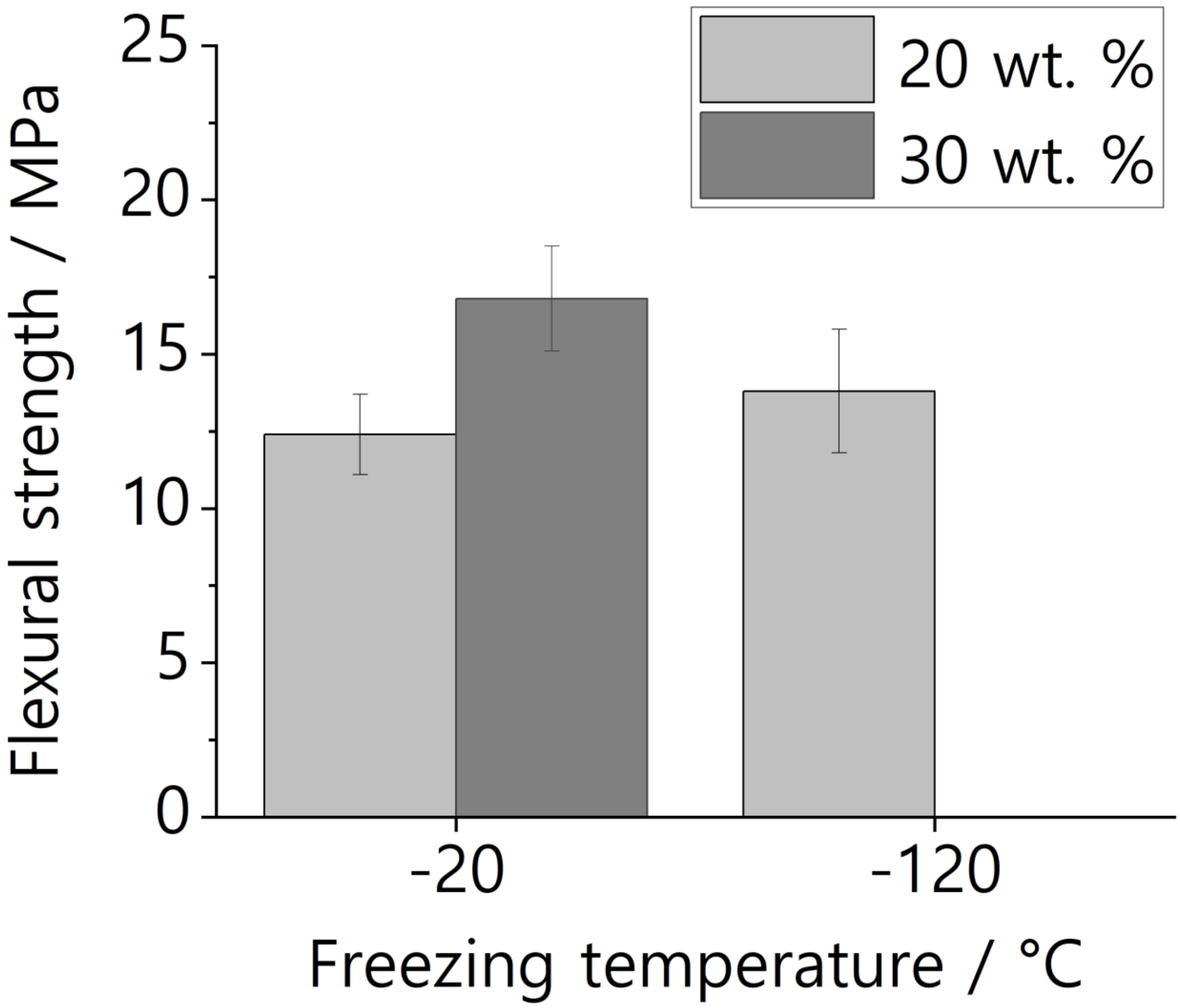

Figure S2 Values of flexural strength from 3-point bending tests following DIN EN 843- 1 for samples with 20 wt% (light blue bars) and 30 wt% (dark blue).

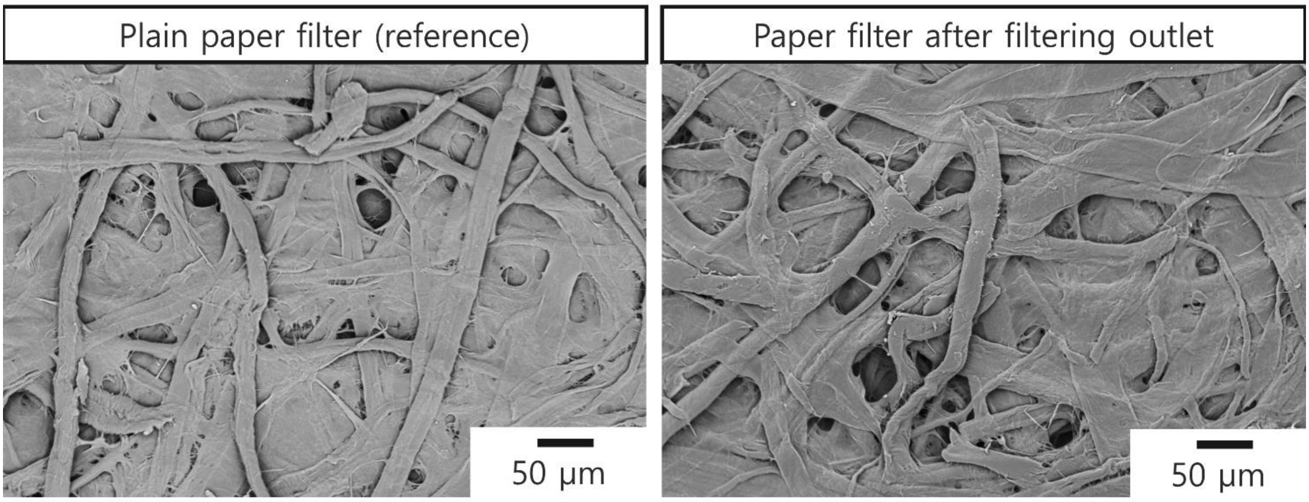

Figure S3 SEM images of paper filter before and after filtering the liquid in the outlet of the gas-liquid phase separation.

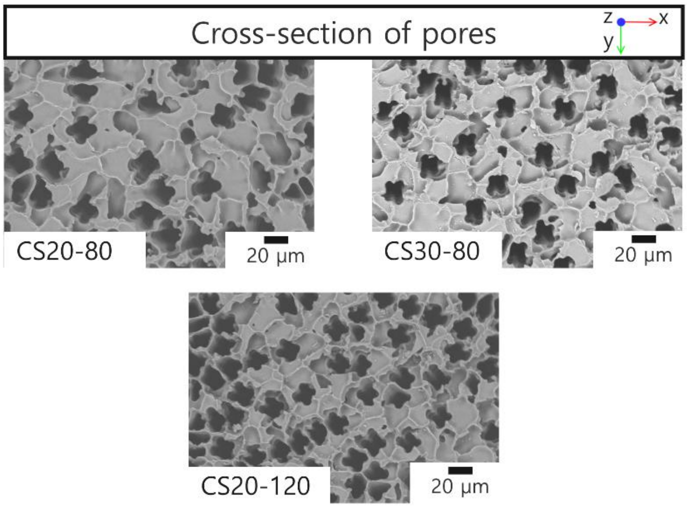

Figure S4 SEM pictures with higher resolution of pore structures with relatively smaller pore window sizes (CS20-80; CS20-120 and CS30-80). The pore morphology is shown from the center position of the samples. Images of cross-section and lateral view with pores perpendicular and parallel to the freezing direction, respectively.
